# Correlation Between Hand Grip Strength and Glycemic Control Among Saudi Children with Chronic Type 1 Diabetes Mellitus (T1DM)

**DOI:** 10.1900/RDS.2023.19.86

**Published:** 2023-06-30

**Authors:** Mohammad H Al-Qahtani, Heba MY El-Basatiny, Fai A. AlQahtani, Shahad A. AlHazzaa, Mohammed Hamdan Hashem, Rana A. Al Balwi, Amani K. Alrowished, Abdullah A Yousef, Alaa A. Aldajani, Bassam H. Awary, Mohammed A. Al Ghamdi, Yara M. Hejazi, Fadhel M. Alfayez

**Affiliations:** 1 1Department of Pediatrics, College of Medicine, King Fahd Hospital of the University, Imam Abdulrahman Bin Faisal University, Dammam, Saudi Arabia,; 2 Department Department of Physical Therapy for Pediatrics, Faculty of Physical Therapy, Cairo University, Elgyza, Egypt,; 3 Physical Education Department, King Fahd University of Petroleum & Minerals, Saudi Arabia.

**Keywords:** handgrip strength, glycemic control, diabetes mellitus

## Abstract

**Objectives:**

We aimed to study the correlation between the hand grip strength as an indicator of the musculoskeletal affection and the degree of glycemic control in Type 1 Diabetic pediatric patients.

**Methods:**

Cross-sectional interventional study conducted among children having chronic T1DM recruited from the pediatric diabetes clinic at King Fahd hospital of the University, Saudi Arabia, they were divided into 3 groups according to their HbA1c level to well controlled, fairly controlled and poorly controlled. Anthropometrics measure taken then handgrip strength for both dominant and nondominant hands were measured using valid and reliable digital JAMAR PLUS hand dynamometer, data collection was performed according to the American Society of Hand Therapists (ASHT) guidelines.

**Results:**

Total of 150 patients, aged 5-18 years, with 56% females and 44% males, two third of them were poorly controlled. Well controlled group showed better hand grip strength than the other 2 groups however it was not statistically significant.

**Conclusions:**

The handgrip strength in Type 1 DM children is affected by the degree of the glycemic control and it might give a clue of early musculoskeletal functional derangement by the effect of chronic hyperglycemia in these affected children.

## 1 Introduction

Diabetes Mellitus (DM) consists of a heterogenous group of disorders characterized by the presence of hyperglycemia due to the inability of the human body to metabolize glucose properly [1]. Type 1 Diabetes Mellitus (T1DM) occurs as a result of chronic insulin deficiency due to the destruction of pancreatic beta cells by an aberrant autoimmune response [2]. The onset of T1DM is influenced by multiple genetic and environmental factors [3]. However, the varying viral and nutritional factors across the globe may influence the clinical appearance of T1DM [4]. Type 2 Diabetes Mellitus, on the other hand, results from the development of insulin resistance [1]. DM is considered a global health burden with a rapidly rising incidence and prevalence. As per the 8th edition of Diabetes Atlas, 35,000 children and adolescents in Saudi Arabia suffer from T1DM, which ranks Saudi Arabia as the 8th country in terms of prevalence of T1DM and the 4th country in terms of incidence with a rate per 100,000 individuals [5], whereas the World Organization ranks Saudi Arabia the 7th in prevalence and the 5th in incidence of T1DM [6].

Over the past few decades, vast advancements in the management of T1DM were achieved. Glycosylated hemoglobin A1c (HbA1c) remains an important guide for the initial diagnosis, management, control, and prediction of complications in diabetic subjects [6]. Nevertheless, individuals with uncontrolled T1DM will invariably develop long-term consequences due to microvascular and macrovascular complications such as blindness, renal failure, and cardiovascular disease. Though often overlooked, musculoskeletal system is also affected by T1DM, and adverse outcomes can be anticipated with suboptimal control of the disease [7].

Diabetic skeletal muscle disease is a common clinical condition observed among individuals with T1DM [8]. It is characterized by lower muscle mass, generalized weakness, functional weakness, in addition to an overall reduction in physical capacity [9]. Muscle weakness contributes to the increased risk of physical disability associated with diabetes in children [10]. Impaired muscle strength has been reported in subjects with diabetes as a late complication of severe diabetic peripheral neuropathy (DPN) with motor nerve involvement [11]. However, other studies have indicated that reduced muscle strength, involving the upper body, may occur earlier in the course of diabetes independent of diabetic peripheral neuropathy [12].

According to American Society of Hand Therapists (ASHT), the evaluation of hand function, by measuring grip and pinch strength, reflects the overall strength of the upper limb [13,14]. Hand grip strength (HGS) is the sum of the strength of the flexor muscles against the palm, and used to evaluate the maximum static force a hand can handle [15,16]. Handheld dynamometry (HHD) provides a simple, inexpensive and versatile alternative for assessing muscle strength [17]. Moderate to good reliability was found across all patient groups with reliability coefficients ranging from 0.80 to 0.99 [18]. In addition to its use to assess disease severity and evaluate the effectiveness of certain interventions, HGS can reflect the general health and level of physical activity of the individual, predicting the overall strength [19-21]. An accumulating body of literature has documented a decline in HGS among individuals with DM, compared with healthy individuals with normal glucose tolerance [22-24], reflecting the link between metabolic and mechanical functions of the muscle [24,25].

Despite the high prevalence of T1DM and the importance of hand function in the activities of daily living of individuals, there is insufficient data concerning the association between glycemic control and hand strength among diabetic children. Therefore, the present study aimed to investigate the correlation between strength measurement through HGS and glycemic control amongst Saudi children diagnosed with T1DM aged between 5 and 18 years with good glycemic control in comparison to children with poor glycemic control following in a pediatric diabetes clinic at King Fahad Hospital of the University in Khobar, Saudi Arabia.

## 2 Materials and methods

### 2.1 Design and subjects

A cross-sectional study was conducted among children diagnosed with T1DM recruited from the pediatric diabetes clinic at King Fahd hospital of the University, Imam Abdulrahman Bin Faisal University in Khobar, Saudi Arabia. 150 children with T1DM were initially screened and assessed to determine age, diagnosis, and inclusion and exclusion criteria. Children aging between 5 and 18 years with a confirmed diagnosis of chronic T1DM for at least 2 years, not known to have another medical condition such as hypertension, anemia, neurological, cardiopulmonary, or renal diseases, and not taking regular medications beside insulin were included in the study. Patients with any other disease affecting their physical activity level, diagnosed with other types of DM, upper limb pain, trauma or fracture around the hand within the past year, severe social deprivation and any history of known psychiatric disease or treatment and mental impairment, that might interfere with their response based on parent report were excluded from the study. All participants were apparently healthy, cognitively competent and able to understand and follow instructions with no evidence of musculoskeletal injuries.

### 2.2 Ethical approval

Ethical approval for this study was granted by the Institutional Review Board at Imam Abdulrahman bin Faisal University and issued the approval number (IRB-2022-01-184). Parents signed the consent form authorizing the child’s participation. The study was conducted in accordance with the Helsinki Declaration of 1975, as revised in 1996 (World Medical Association, 1996).

### 2.3 Biochemical measurements

The degree of control of diabetes was evaluated by measuring the average of the last 3 readings of glycosylated haemoglobin (HbA1C, HPLC method). Blood samples were collected by a trained paediatric nurse.

### 2.4 Anthropometric parameters

Participants’ weight and height were measured, and the body mass index (BMI) was calculated as Weight (W) in kg / [Height (H) in meter]^2^. Medical conditions of the participants, such as the T1DM chronicity, appropriate medication doses and HbA1c, were assessed by a pediatric endocrinologist in the clinic. Participants were assessed physically and clinically by a pediatrician, and according to their HbA1c they were categorized into three groups: group (A) with poor glycemic control with an HbA1c above 8.5%, group (B) with fairly controlled DM with an HbA1c between 7% and 8.5%, and group (C) with well controlled DM with an HbA1c of 7%. All groups were matched by average age, weight, height, and BMI. All participants have received the same verbal cues and directions for outcome measurement.

### 2.5 Handgrip strength assessment

Under direct supervision of an expert physical therapy faculty member, handgrip strength for both dominant and nondominant hands were measured using valid and reliable digital JAMAR PLUS hand dynamometer (Patterson Medical, Sammons Preston, Jackson, MI). Data collection was performed according to the American Society of Hand Therapists (ASHT) guidelines [26]. The participants were seated on a chair without armrests with their feet flat on the floor, shoulder was abducted and neutrally rotated, elbow flexed at 90°, forearm in a neutral position, and wrist between 0 and 30° extension and between 0 and 15° ulnar deviation. Testing positions demonstrations and verbal instructions were provided. The participants received verbal encouragement to squeeze the gauge as hard as possible to exert their maximal force during each trial and hold for 5 seconds. Each test was performed three times to collect HGS data, and the average was calculated and expressed in kilograms. If a measurement displayed a difference of over 10% from the previously achieved measurements, it would lead to performing a fourth trial [26,27]. The measurements of HGS was done in alternating order between the dominant and nondominant hands, with a 1-min rest between them to minimize fatigue effect [28, 16]. The calibration of Jamar hand dynamometer was tested periodically during the study [16].

### 2.6 Statistical analysis

The data analysis was performed using SPSS version 26(Armonk, NY: IBM Corp. USA). The normality of the data distribution was assessed using KolmogrovSmirnov test which showed normal data distribution if p > 0.05. Continuous variables were expressed as mean ± SD (for variables with normal distributions) or medians (interquartile range) (for variables with nonnormal distributions, and categorical variables were expressed as frequency and percentages (%). The demographic and baseline characteristics, between levels of glycemic control were compared using the Chi square test or Fisher’s exact test for categorical variables, ANOVA/ Kruskal-Wallis tests for continuous variables, as appropriate for more than two groups. Student’s t-test or Mann-Whitney U test was used to compare the dexterity and the various characteristics whichever was appropriate. Spearman’s rank correlation coefficient was used to analyse the relationship between the hand grip strength with duration of diabetes and also with BMI. Two-way ANOVA was used to test for the differences between the levels of glycemic control and hand dexterity and also its interaction effect in the assessment of hand grip strength. A p-value of < 0.05 was considered significant for the analysis.

## 3 Results

Details of the characteristics of the study participants are presented in Table 1. A total of 150 children with age range, 5 to 18 years participated in the study. Among the 150 (66 males, 84 females) participants included in the study, duration of diabetes was 5.0 years (interquartile range 3.0– 7.0 years). With poorly controlled glycemic level was found in 103 (68.7%) participants, fairly controlled in 31 (20.7%) participants, and well controlled in 16 (10.7%) of the participants. The mean±SD for BMI was 20.20±4.5 kg/m2. The right- and left-hand dominance was found in 139 (92.7%) and 11 (7.3%) of the participants, respectively. No significant difference was found in the proportion of participants with different level of glycemic control in all the demographic and clinical characteristics, except for weight and those variables related to glucose level.

**Table 1.** Baseline characteristics of participant

Variables	Total (n = 150)	Glycemic control			P value
		Poorly-Controlled (n = 103)	Fairly-Controlled (n = 31)	Well controlled (n = 16)	
**Sex**					
Male	66 (44.0)	46 (44.7)	11 (35.5)	9 (56.3)	0.386
Female	84 (56.0)	57 (55.3)	20 (64.5)	7 (43.8)	
**Age**	12.00 (10.0,14.3)	13.00 (11.0,14.0)	11.00 (10.0,15.0)	10.00 (8.0,12.8)	0.064
**BMI** mean ± SD	20.20±4.5	20.64±4.7	19.73±4.3	18.27±2.5	0.119
**Dominant side**					
Right	139 (92.7)	96 (93.2)	29 (93.5)	14 (87.5)	0.623*
Left	11 (7.3)	7 (6.8)	2 (6.5)	2 (12.5)	
**Duration of DM**	5.00 (3.0,7.0)	5.00 (2.0,7.0)	5.00 (3.0,8.0)	2.00 (1.0,4.0)	0.012
**HBA1C level**	10.20 (9.0,12.0)	11.00 (10.1,12.5)	8.80 (8.5,9.1)	7.48 (6.9,7.9)	0.000
**Systolic** mean ± SD	113.53±2.9	114.46±11.6	112.19±11.0	110.19±10.8	0.294
**Diastolic**	73.25±8.6	73.00 (68.0,80.0)	72.00 (68.0,77.0)	73.00 (68.0,80.0)	0.124
**Heart rate**	92.00 (85.8,104.0)	92.00 (85.8,104.0)	92.00 (85.8,104.0)	71.00 (65.8,73.5)	0.893
**Respiratory rate**	17.00 (14.0,20.0)	15.00 (14.0,20.0)	18.00 (14.0,20.0)	15.50 (14.0,18.0)	0.286
**Dominant side 1st trial**	11.00 (8.0,16.0)	11.00 (8.0,16.0)	11.00 (7.0,17.0)	9.00 (7.3,11.8)	0.364
**Dominant side 2nd trial**	10.00 (8.0,17.0)	10.00 (8.0,17.0)	10.00 (8.0,17.0)	9.50 (6.3,12.8)	0.608
**Dominant side 3rd trial**	11.00 (8.0,15.0)	10.00 (8.0,15.0)	10.00 (8.0,16.0)	9.00 (5.3,13.5)	0.577
**Mean Dominant side**	10.60 (8.0,15.39)	10.60 (8.0,15.7)	11.0 (7.6,16.6)	9.15 (6.6,12.7)	0.465
**Non-dominant side 1st trial**	10.00 (8.0,14.3)	10.00 (8.0,15.0)	10.00 (7.0,17.0)	9.00 (7.0,12.0)	0.656
**Non-dominant side 2nd trial**	9.00 (6.8,14.0)	9.00 (7.0,14.0)	10.00 (6.0,16.0)	9.00 (6.0,11.5)	0.581
**Non-dominant side 3rd trial**	9.00 (6.8,14.0)	9.00 (7.0,14.0)	10.00 (5.0,15.0)	8.00 (6.0,12.0)	0.703
**Mean Non-dominant side**	9.60 (6.9,13.1)	9.60 (7.3,13.0)	9.60 (5.3,15.3)	8.60 (6.2,12.3)	0.594

Categorical variables are expressed as number (%), continuous Data are expressed as the mean ± standard deviation or median (inter quartile range); * - Fisher’s exact test

Table 2 shows the median (interquartile range) of the HGS for the different levels of glycemic control for the dominant and non-dominant hands. The results revealed that individuals with well controlled gylcemic level had the best performance in both hands, but the difference was not significantly different from the participants with other levels of glycemic control. The proportion of males were significantly higher in left hand dominance with 81.8% males compared to 41.0% males in the right-hand dominant group. All the other variables were not significantly different between right- and left-hand dominance. (See Table 3)

**Table 2.** Relationship between handgrip strength with glycemic control for the dominant and the non-dominant hand of the participants (n=150)

Variable	Glycemic control			KW H#	P value
	Poorly-Controlled (n = 103)	Fairly-Controlled (n = 31)	Well controlled (n = 16)		
Dominant hand	10.30 (8.0,16.6)	10.60 (6.6,15.3)	9.15 (6.6,12.7)	1.640	0.441
Non-dominant hand	9.60 (7.3,12.7)	10.00 (5.3,16.6)	8.60 (6.2,12.3)	0.998	0.607

#- Kruskal Wallis test statistic

**Table 3.** Distribution of baseline and clinical characteristics of the participants

Variables	Right hand (n = 139)	Left hand (n = 11)	P value
**Sex**			
Male	57 (41.0)	9 (81.8)	0.011*
Female	82 (59.0)	2 (18.2)	
**Age**	12.00 (10.0,14.0)	14.00 (11.0,15.0)	0.543
**BMI** mean ± SD	20.37±4.6	18.09±2.5	0.107
**Glycemic control**			
Poorly-Controlled	96 (69.1)	7 (63.6)	0.623*
Fairly-Controlled	29 (93.5)	2 (18.2)	
Well controlled	14 (10.1)	2 (18.2)	
**Duration of DM**	5.00 (2.0,7.0)	4.00 (2.0,8.0)	0.811
**HBA1C level**	10.3 (9.1,12.0)	10.00 (8.5,13.0)	0.945
**Systolic mean** ± SD	113.22±11.5	117.45±10.4	0.239
**Diastolic mean** ± SD	73.35±8.7	72.0±6.2	0.616
**Heart rate**	93.00 (85.0,105.0)	87.00 (86.0,95.0)	0.090
**Respiratory rate**	18.00 (14.0,20.0)	14.00 (14.0,18.0)	0.146
**HG RT side 1st trial**	10.00 (8.0,17.0)	12.00 (10.0,13.0)	0.400
**HG RT side 2nd trial**	10.00 (8.0,17.0)	11.00 (10.0,13.0)	0.758
**HG RT side 3rd trial**	10.00 (8.0,15.0)	10.00 (9.0,14.0)	0.957
**Mean HG RT side**	10.6 (8.0,16.3)	11.30 (9.3,12.3)	0.686
**HG LT side 1st trial**	10.00 (7.0,15.0)	11.00 (9.0,14.0)	0.188
**HG LT side 2nd trial**	9.00 (6.0,14.0)	10.00 (8.0,16.0)	0.625
**HG LT side 3rd trial**	9.00 (6.0,14.0)	11.00 (10.0,15.0)	0.105
**Mean HG LT side**	9.30 (6.6,13.0)	10.00 (7.3,15.3)	0.708

Table 4 presents the HGS of the participants measured on both hands are compared between the dominant and non-dominant hands. For the right hand, a median of 10.60 (inter quartile range 8.0,16.3) was measured for the right dominant hand while the same was 11.30 (9.3,16.6) in the non-dominant hand (p=0.686). Similarly, for the left hand, a median of 10.00 (inter quartile range 7.3,15.3) was measured for the left dominant hand while the same was 9.30 (6.6,13.0) in the non-dominant right-hand group (p=0.708) (Table 5).

**Table 4.** Comparison of handgrip strength measured on each hand

Variable	Grip strength measured on	
	Right hand	Left hand
Dominant hand	10.60 (8.0,16.3)	10.00 (7.3,15.3)
Non-dominant hand	11.30 (9.3,16.6)	9.30 (6.6,13.0)
**P value**	0.686	0.708

**Table 5.** Handgrip strength as a function of glycemic control

Hand	Effect	df	F	P	η2
**Right**	Glycemic control	2,144	0.924	0.399	0.013
	Dominant side	1,144	0.003	0.953	0.000
	Glycemic control*Dominant side	2,144	0.687	0.505	0.009
**Left**	Glycemic control	2,144	0.983	0.377	0.013
	Dominant side	1,144	0.233	0.630	0.002
	Glycemic control*Dominant side	2,144	0.624	0.537	0.009

**Figure 1:**
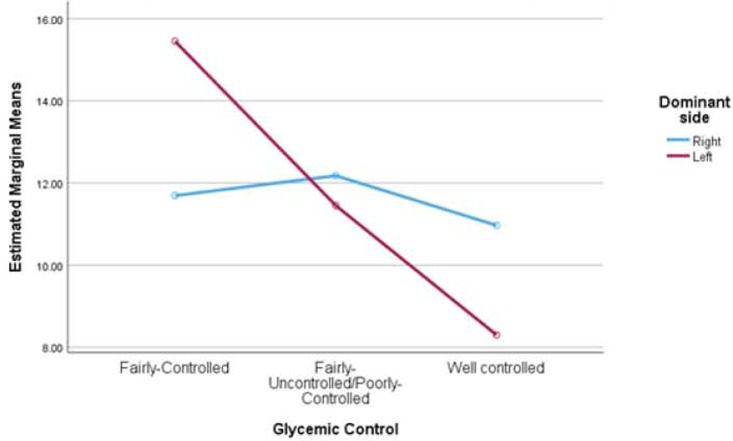
Estimated Marginal Means of Hand Grip strength of the right hand

**Figure 2:**
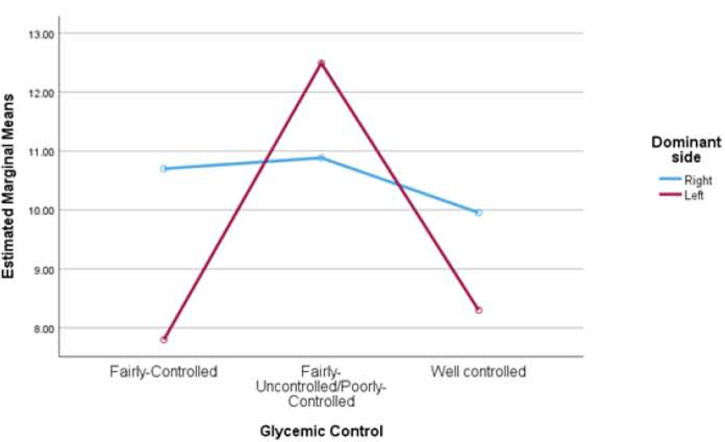
Estimated Marginal Means of Hand Grip strength of the left hand

The results revealed that the participants did not show a statistically significant difference in HGS measured in the right hand for glycemic control, dominance as well as for the interaction of the two [F(2,144) = 0.924, p = 0.399, η2 = 0.013], [F(1,144) = 0.003, p = 0.953, η2 = 0.000] and [F(2,144) = 0.687, p = 0.505, η2 = 0.009], respectively. Similarly, for the left hand, HGS measured for glycemic control, dominance as well as for the interaction of the two was not statistically significant [F(2,144) = 0.983, p = 0.377, η2 = 0.013], [F(1,144) = 0.233, p = 0.630, η2 = 0.002] and [F(2,144) = 0.6245, p = 0. 537, η2 = 0.009]. Also see figure 1 and 2.

Table 6 shows the correlation between age and HGS with each glycemic control group. The correlations showed a positive linear correlation, and the strength of the correlation coefficient was moderate and was statistically significant except for the correlation of HGS of the left hand in the well-controlled glycemic group. From table 7, we see that from age 11 and above, both the sexes were not significantly different from the normative hand grip strength taken from the reference study.

**Table 6.** Correlation between age and handgrip strength on each hand for each glycemic control groups

Variable	Age correlated with Grip strength measured on			
	Right hand		Left hand	
	r/rho	P value	r/rho	P value
**Glycemic control**				
Poorly-Controlled	0.481	0.000	0.414	0.000
Fairly-Controlled	0.571	0.001	0.560	0.001*
Well controlled	0.571	0.021*	0.473	0.063*

* - Pearson’s correlation

**Table 7.** Comparison of handgrip strength of both sexes with normative levels from a reference study

Hand grip strength						
		Reference study		Current study		
Age	Sex	N	Mean±SD	N	Mean±SD	Pvalue
5	Male			2	8.0±2.8	-
	Female					-
6	Male	14	8.5±6.1			-
	Female	14	8.5±5	1	6.0	-
7	Male	61	18.6±10.5	4	8.4±1.5	0.000
	Female	37	13.3±5.8	5	6.1±1.2	0.000
8	Male	22	15.8±9.6	7	7.6±3.1	0.000
	Female	23	13.5±6.5	6	6.8±1.0	0.000
9	Male	31	18.7±9.7	3	14.3±7.8	0.438
	Female	31	12.5±6.3	1	6.0	-
10	Male	15	15.1±9.8	4	7.2±3.0	0.016
	Female	19	12.7±5.1	11	8.4±2.9	0.006
11	Male	27	12.4±4.8	10	11.2±2.7	0.348
	Female	26	12.9±5.7	5	10.0±3.3	0.15
12	Male	31	17.4±6.6	5	12.0±4.9	0.069
	Female	22	12.0±6.5	14	13.1±5.4	0.586
13	Male	29	17.8±9.5	10	15.5±4.9	0.335
	Female	20	13.4±6.4	6	12.0±3.6	0.505
14	Male	23	16.6±8.0	4	15.0±5.5	0.638
	Female	26	12.5±6.7	15	12.0±4.1	0.769
15	Male	23	14.5±8.2	9	16.6±7.0	0.478
	Female	28	13.2±5.1	11	15.5±5.3	0.234
16	Male	19	14.1±8.8	5	17.0±5.7	0.394
	Female	25	12.4±6.2	5	9.1±2.2	0.051
17	Male	16	16.6±8.1	3	24.4±6.9	0.175
	Female	15	12.8±4.6	3	11.2±6.9	0.732
18	Male	7	14.5±6.8			-
	Female	12	14.5±6.8	1	10.60	-

**Figure 3:**
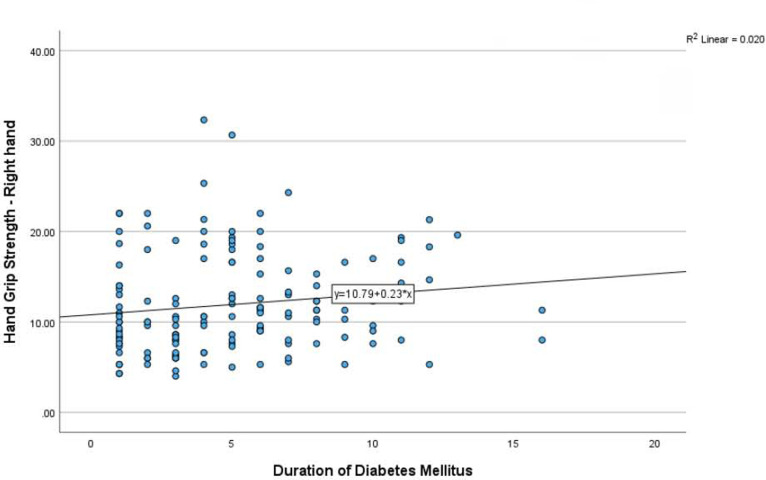
Relationship between duration of diabetes and HGS-Right hand

**Figure 4:**
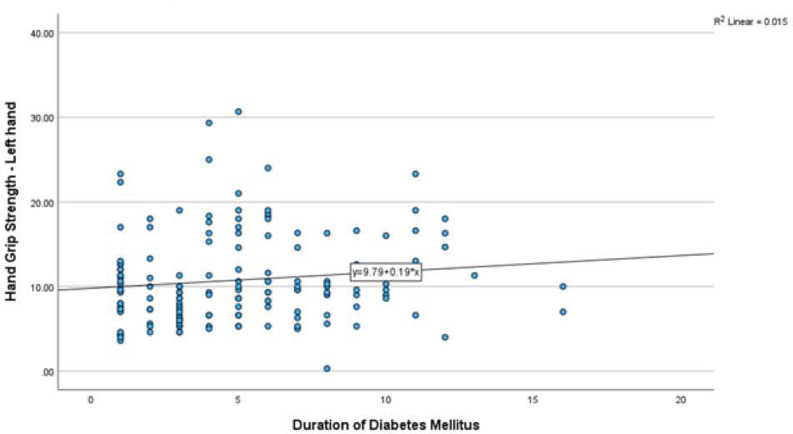
Relationship between duration of diabetes and HGS-Left hand

The relationship between the duration of diabetes with hand grip strength of the right hand showed the spearman’s rank correlation coefficient was low with ρ = 0.204 p=0.012. similarly, the relationship between the duration of diabetes with hand grip strength of the left hand showed the Spearman’s rank correlation coefficient was low with ρ = 0.164 p=0.045. Also see figure 3 and 4.

**Figure 5:**
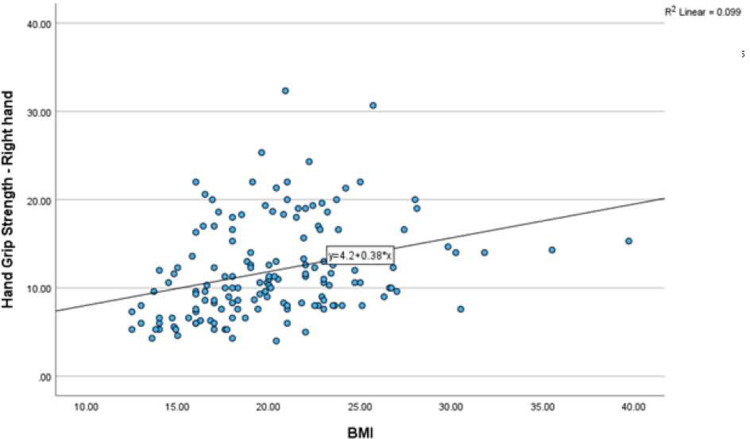
Relationship between BMI and HGS-Right hand

**Figure 6:**
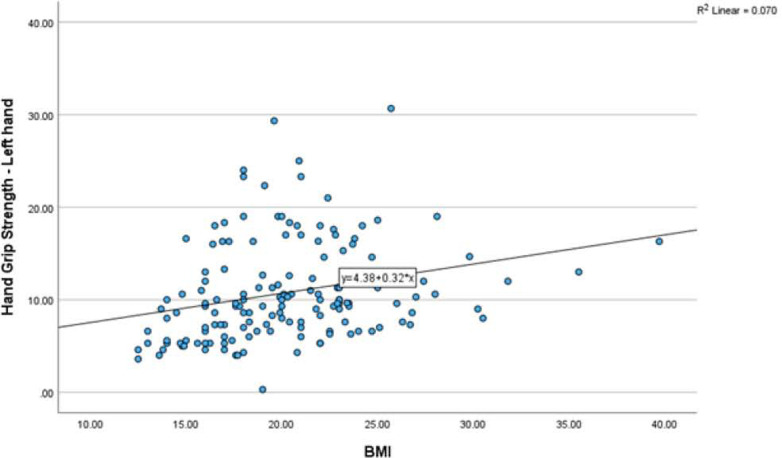
Relationship between BMI and HGS-Left hand

The relationship between the BMI with hand grip strength of the right hand showed the Spearman’s rank correlation coefficient was low with ρ = 0.395 p=0.000. similarly, the relationship between the duration of diabetes with hand grip strength of the left hand showed the Spearman’s rank correlation coefficient was low with ρ = 0.350 p=0.000. Also see figure 5 and 6.

## 4 Discussion

The present study has investigated the correlation between T1DM and HGS among Saudi children aged between 5 and 18 years with different levels of glycemic control. No significant differences in HGS were observed between patients with poorly controlled disease in comparison to those with fairly controlled and well controlled T1DM. Nonetheless, although it was statistically insignificant, participants with well controlled T1DM have shown the best performance in both dominant and non-dominant hands compared to children with other levels of glycemic control. Consistent with our findings, Dongare-Bhor et al. reported a lack of correlation between HGS and HbA1C [29]. In adults with T1DM, Wallymahmed et al. reported a significant negative correlation between HGS and HbA1C which opposes the results of the current study [30]. Fricke et al., in contrast, reported a significantly higher HGS in children with an HbA1C above 8.5% compared to those with lower values of HbA1C [31].

In addition, we report a significantly low correlation between HGS and disease duration. In line with our findings, Dongare-Bhor et al. reported the lack of correlation between HGS and disease duration across diabetic children who had the disease for more than 5 years compared to those who had it for shorter durations [29]. Klara et al., on the contrary, evaluated the dynamic muscle function using jumping mechanography and reported a decline in muscle function among adolescents with T1DM which was particularly evident in patients who had T1DM longer than 9 years [32]. The previous study has anticipated a further deterioration in muscle function in adulthood with increased T1DM duration [32]. Reduced HGS and impaired muscle function among adults was reported by several studies to be a complication of T1DM and T2DM especially in patients with DPN and carpel tunnel syndrome [32-34].

A recently published community-based study conducted by Alqahtani et al. measured the HGS among 616 Saudi children aged between 6 and 18 years old across different cities in Saudi Arabia [35]. The reported normative values of HGS have been used as a reference for comparison in the present study. Children with T1DM aged 7 to 10 years from both sexes were found to have a significantly lower HGS compared to the reference population except for males aging 9 years. However, no significant difference in HGS was found between the reference population and diabetic children aged 11 years and above.

Fricke et al. found a significantly lower maximal isometric force among children with T1DM in all age groups compared to age-matched reference counterparts [31], which is partially congruent with the findings of the current study. Similarly, DongareBhor et al. reported that children with T1DM had a significantly lower HGS compared to the controls [29]. Bechtold et al., in contrast, have investigated the impact of T1DM on the development of bone and muscle and reported a significantly higher HGS among diabetic children in comparison to controls which was attributed to intensive motivation of the participants during the assessment [36].

Although knowledge is currently limited and the existing evidence remains equivocal, multiple postulations describing the association between decreased muscle function and T1DM have been proposed. The effect of T1DM on skeletal muscle function is thought to be multifactorial with altered levels of hormones and hyperglycemia being the major contributors [8,32]. Insulin is a potent anabolic hormone that promote protein synthesis and inhibits the degradation of protein in the skeletal muscle [8,37]. A deficiency in insulin leads to a protein catabolic state that results in the loss of muscular tissue [32], which could be a possible underlying mechanism that leads to impaired muscle function in diabetic individuals. In addition, several studies found that Insulin-like Growth Factor-1 (IGF-1), an essential growth factor of the skeletal muscle, was reduced in adolescents and adults with T1DM, raising the suggestion that it may have a role in muscle growth impairment in T1DM [8].

Furthermore, in a long-standing hyperglycemic state, protein glycation occurs in skeletal muscle, a process at which proteins undergo chemical modification as a result of sugars reduction [8,32].) In early stages of this reaction, myosin motility is found to be reduced [32]. Further oxidation reactions result in advanced glycation end-product (AGE), the contribution of which in T1DM-related complications has been clearly established [8]. In skeletal muscle, the accumulation of AGE was suggested by several studies to be associated with a decline in muscle function in adult patients with T1DM, T2DM and elderly individuals [38].

In the present study, a significant positive linear correlation was observed between age and HGS among diabetic children. Similarly, Bechtold et al. found that HGS has increased significantly with age in children. In accordance with previous findings, an overall trend of increasing HGS with age among healthy children was reported by several studies [35,39]. This increase is probably attributed to the physiological changes and development in muscle strength of the upper limbs in both genders with age [35].

Although the correlation between HGS and T1DM remains uncertain with heterogenous findings [29,30,31,36], chronic diseases are often associated with deterioration in muscle function [31]. Several studies have reported a decrease in HGS among pediatric and adult patients suffering from chronic conditions [31,40,41]. Rauch et al. reported reduced maximal isometric grip force among children and adolescents with cystic fibrosis and kidney transplantation [40]. In adults, HGS was found to approximately 25-50% lower in patients on hemodialysis compared to general population [41]. In addition, HGS weakness was found to be associated with multimorbidity including chronic diseases such as anemia, stage 3 of chronic kidney disease or above, stroke, and kyphosis [42]. Hence, further research is imperative to assess the impact of T1DM on muscle growth in children and adolescents to facilitate early intervention and prevent long-term complications affecting the quality of life of individuals.

### 4.1 Limitations

We have some limitations to be considered in interpreting the results of our study; first, this study is cross-sectional with relatively small sample sizes, dealing with single center of the country. However, this study provided a baseline assessment based on grip strength in Type 1 diabetic children, given the scarcity of data on national as well international paediatric population,therefore, comprehensive studies and innovative research are strongly needed and the clinical trials must be well designed, adequately powered, carefully controlled, cautiously conducted, and multicenter approaches in order to prevent or delay the development of the devastating musculoskeletal complications of the T1DM in pediatric age group.

Second, the study compared the controlled type 1 diabetic children, determined by average A1c level compared to poorly controlled diabetic children, not able to have normal matching children as a baseline comparison since it is difficult to recruit normal children from the pediatric clinics in hospital population, however we compared them to the single Saudi published reference standard.

### 4.2 Conclusion

Chronic standing uncontrolled type 1 diabetes mellitus has significant impact on all the body systems including the musculoskeletal system, which might be detected early by performing some standardized motor ability tests, which will help in prevention and early management of such complications.

## Abbreviations

DM: Diabetes Mellitus

T1DM: Type 1 diabetes Mellitus

DNP: Diabetic Neuropathy

ASHT: American Society of Hand Therapists

HGS: Hand Grip Strength

HHD: Handheld dynamometry

HbA1c: Glycosylated Hemoglobin A1c

BMI: Body Mass Index
